# Health anxiety and illness-related fears across diverse chronic illnesses: A systematic review on conceptualization, measurement, prevalence, course, and correlates

**DOI:** 10.1371/journal.pone.0234124

**Published:** 2020-07-27

**Authors:** Sophie Lebel, Brittany Mutsaers, Christina Tomei, Caroline Séguin Leclair, Georden Jones, Danielle Petricone-Westwood, Nicole Rutkowski, Viviane Ta, Geneviève Trudel, Simone Zofia Laflamme, Andrée-Anne Lavigne, Andreas Dinkel

**Affiliations:** 1 School of Psychology, University of Ottawa, Ottawa, Ontario, Canada; 2 Department of Psychosomatic Medicine and Psychotherapy, Klinikum rechts der Isar, School of Medicine, Technical University of Munich, Munich, Germany; Technion Israel Institute of Technology, ISRAEL

## Abstract

**Background:**

Patients with chronic diseases commonly report fears of illness or symptoms recurring or worsening. These fears have been addressed from an illness-specific perspective (e.g., fear of cancer recurrence), a generic illness perspective (e.g., fear of progression), and a psychiatric perspective (DSM-5 illness anxiety disorder and somatic symptom disorder). The broader concept of health anxiety (HA) can also be applied to patients with a chronic disease. This review was conducted to investigate the conceptual, theoretical, measurement-overlap, and differences between these distinct perspectives. We also aimed to summarize prevalence, course, and correlates of these fears in different chronic illnesses.

**Methods:**

We used PsycINFO, PubMED, CINAHL, Web of Science, SCOPUS, and PSYNDEX to conduct a systematic review of studies pertaining to these fears in chronic illness published from January 1996 to October 2017. A total of 401 articles were retained.

**Results:**

There were commonalities across different conceptualizations and diseases: a high prevalence of clinical levels of fears (>20%), a stable course over time, and a deleterious impact on quality of life. Reviewed studies used definitions, models, and measures that were illness-specific, with only a minority employing a psychiatric perspective, limiting cross-disease generalizability. There appears to be some applicability of DSM-5 disorders to the experience of fear of illness/symptoms in patients with a chronic illness. While conceptualizing HA on a continuum ranging from mild and transient to severe may be appropriate, there is a lack of agreement about when the level of fear becomes ‘excessive.’ The definitions, models, and measures of HA across chronic illnesses involve affective, cognitive, behavioral, and perceptual features.

**Conclusions:**

The concept of HA may offer a unifying conceptual perspective on the fears of illness/symptoms worsening or returning commonly experienced by those with chronic disease.

## Introduction

### Rationale

Chronic diseases are long-term conditions that develop slowly and increase in severity over time. Most often, chronic diseases are incurable, and treatment is focused primarily on the management of symptoms [1]. Chronic diseases account for 60% of all deaths and 43% of the global burden of disease [2]. Those with chronic conditions must cope with the persistence and the unpredictability of their illness. They often report feeling anxious and worried about their condition or its symptoms recurring or worsening. This experience was aptly described by cancer survivors who likened their worry about cancer recurrence to the sword of Damocles that hangs over them for the rest of their lives [3]. As the population ages, and chronic diseases become increasingly prevalent, there is a growing body of literature examining the emotional state of chronically ill patients [3–8].

The fear and worry that occurs in response to living with a chronic illness has been called health anxiety (HA) [4–9]. Generally, HA arises when bodily sensations or changes are believed to be indicative of serious illness [10]. HA involves affective, cognitive, behavioral, and perceptual features [11]. More specifically, HA consists of distressing emotions (e.g., fear), thoughts of danger, and physiological arousal [12]. The experience of HA varies across individuals, and has therefore been conceptualized on a continuum ranging from mild and transient to severe and chronic [12]. In the context of chronic illness, there is evidence that moderate to high levels of HA are highly prevalent. For example, a systematic review suggested a prevalence of 49% for moderate to high fear of cancer recurrence among cancer survivors, where cancer is considered to be a chronic illness [13]. This review also found mixed results for the intensity of fear of cancer recurrence over time. Some studies suggested that the fear did not change, while others showed that it was highest after cancer diagnosis and treatment, followed by a decrease to a more stable level of intensity [13]. Few sociodemographic and medical predictors were identified by this review, with the exception of younger age and presence of somatic symptoms. Illness- and treatment-related factors seem to be of minor importance. Thus much work remains to identify individuals with a chronic illness who are most at risk of presenting with elevated HA.

As there are numerous chronic illnesses that involve a variety of symptom presentations, research on HA tends to be disease specific (i.e., fear of cancer recurrence/progression [14], fear of hypoglycemia [15], cardiac anxiety [16] etc.). Within each chronic illness, researchers have examined disease-specific worries and have developed measures and interventions for each of these manifestations. We refer to this approach as the disease- or symptom-specific perspective. This narrower view of HA in chronic illnesses has resulted in fairly isolated areas of research with limited cross-disease applicability in terms of models and treatments. A systematic review of interventions for HA found evidence of effective interventions for most of these specific chronic illnesses. However, these were very diverse, ranging from pragmatic interventions that directly aim to reduce the risk of health problems in people with diabetes, to psychotherapeutic interventions targeting mechanisms of action (e.g., intolerance of uncertainty) underlying HA in cancer survivors. Few types of interventions were measured across diseases [17], limiting generalizability of findings. Developing an understanding of HA across chronic illnesses (rather than within specific diseases) would help to address this gap. Importantly, the Diagnostic and Statistical Manual of Mental Disorders [18] characterizes HA more generally, which differs from research that predominantly looks at presentations of HA within each chronic illness separately. We refer to this broader definition that relies on DSM-IV or DSM-5 concepts as the psychiatric perspective.

The meaning and connotations associated with the term “health anxiety” can strongly influence how this concept is understood [9]. For example, HA can be thought of as being synonymous with “hypochondriasis” and regarded as a mental disorder (i.e., the *conviction* of having a physical disease despite negative medical findings) [9]. Alternatively, HA can be considered a normative response to managing a chronic illness where a real threat to one’s health and wellbeing is present [8]. Given the variability in the meaning attributed to HA, significant changes were made to the diagnosis of “hypochondriasis” in the fifth edition of the Diagnostic and Statistical Manual of Mental Disorders (DSM-5) [18].

In the Diagnostic and Statistical Manual of Mental Disorders, 4^th^ Edition (DSM-IV), the concepts of HA and hypochondriasis were used interchangeably and referred to excessive fear or worry about ill health, which is believed to result from a preoccupation with the *incorrect* belief that one has, or is in danger of developing, a serious disease or a medical condition [19] Hypochondriasis has a low reported prevalence of about 1–2% in general population samples [20]. In contrast, the *concern* of becoming or possibly being ill, is more common, with a prevalence rate of about 3% in the general population and 20% in hospital out-patients [20]. With the introduction of the Diagnostic and Statistical Manual of Mental Disorders, 5^th^ Edition (DSM-5) in 2013, hypochondriasis was redefined and replaced with two new concepts: somatic symptom disorder and illness anxiety disorder [18]. Somatic symptom disorder is characterized by somatic symptoms, *whether medically explained or not*, that are either very distressing, or result in noteworthy disruption of functioning. Somatic symptom disorder is also characterized by excessive and disproportionate thoughts, feelings, and behaviors relative to those specific symptoms [18]. Illness anxiety disorder entails a preoccupation with *having or acquiring* a serious medical illness, where somatic symptoms are either absent or mild, and with a specifier of either an avoidant or reassurance seeking presentation [18].

The introduction of these new diagnostic labels in DSM-5 is significant for two main reasons. First, a psychiatric diagnosis can now be applied to individuals experiencing excessive HA who also have a diagnosed physical disease, including chronically ill individuals. Second, this now explicitly unifies the concepts of HA and hypochondriasis, which may be considered an advantage because this could facilitate more consistency in how these terms are used in the literature. These revisions in the DSM-5 have been criticized and include debate over the reliability and validity of the new DSM-5 diagnoses for patients with chronic diseases. The concern is the risk of over diagnosing individuals as “mentally ill” when their fear could be considered realistic given their condition [21, 22].

Although much of the research on HA is disease-specific, the fear of progression has been introduced as a generic, integrative construct applicable across various chronic diseases [23, 24]. Fear of progression provides an alternative to the psychiatric view of HA/hypochondriasis where HA in people with chronic illness is seen to be a *normal*, non-neurotic reaction in the face of a real threat [23].

### Research questions

In summary, researchers and clinicians currently have two very different ways of understanding fears about health/symptoms in people with chronic illnesses, each with advantages and disadvantages. The first is a disease- or symptom-specific perspective and the second is a psychiatric perspective. In order to understand which approach may be most relevant to the study of fears about health/symptoms in chronic illness, we examined two specific questions. How do researchers theoretically conceptualize, define, and measure these fears in their studies of individuals with a chronic disease? What is the prevalence, course, and who are the individuals most likely to be affected by these fears across chronic illnesses?

### Objectives

To answer these questions, we undertook a systematic review of the literature on HA across chronic illnesses with the goal of providing a unified snapshot of research fields that have previously operated in silos. A narrative synthesis of the results was used rather than a statistical approach because of the anticipated heterogeneity in HA constructs, study design, populations, and measures. With this review of fears of illness/symptoms across diseases we are able to suggest a unifying perspective for this very broad field of research and are in a position to recommend specific terminology, a generic definition, and most suitable measures to inform future studies.

## Method

### Search strategy and data sources

The literature search was performed in two phases. During phase I, the following databases were used in September and October 2014: a) PsycINFO, b) PubMED, c) CINAHL, d) Web of Science and e) SCOPUS. These databases were chosen in consultation with our institution librarian to reflect specific fields (e.g. nursing and allied health with CINAHL) as well as a broad perspective (e.g. SCOPUS). To guide our search, we conceptualized HA as *concerns or worry or fear that one’s illness or an aspect of or a symptom of one’s illness (i*.*e*. *hypoglycemia*, *falling) may worsen*, *progress*, *or recur*. The keyword formula was developed based on the targeted chronic illnesses and the respective HA constructs (e.g., for searches on diabetes the HA construct of fear of hypoglycemia was used), as well as general constructs (e.g. fear of illness progression, HA, health concerns). For a complete list of keywords by illness type, please see Table 1.

**Table 1:** Complete list of keywords by illness type used for the database search.

“fear of disease progression” “health worry” OR “health fear” OR “health anxiety” OR “fear of recurrence” AND chronic illness “health worry” OR “health fear” OR “health anxiety” OR “fear of recurrence” AND chronic disease “health worry” OR “health fear” OR “health anxiety” OR “fear of recurrence” AND stroke OR diabetes OR cardi* OR asthma OR epilepsy OR parkinson OR hiv “health worry” OR “health fear” OR “health anxiety” OR “fear of recurrence” AND cancer OR neoplasm “health worry” OR “health fear” OR “health anxiety” OR “fear of recurrence” AND multiple sclerosis OR irritable bowel disease OR arthritis OR chronic kidney disease OR end-stage renal* “cardiac anxiety” “heart-focused anxiety” “fear of hypoglycemia” AND diabetes “fear of shock” AND implantable cardioverter defibrillator^a^ “fear” AND implantable cardioverter defibrillator^a^ “fear of dyspnea” AND (COPD OR asthma) “fear of seizure” AND epilepsy “fear of falling” AND Parkinson “fear of pain” AND cancer OR neoplasm “fear of pain” AND chronic pain^a^

^a^The results of these searches were excluded from the full article review phase

In addition, to identify German-language articles not covered by the included databases, a literature search was conducted using the German database PSYNDEX in July 2015. This database includes literature published in German-speaking countries. Reference lists of relevant German-language publications were also searched. All duplicates were removed once the searches were complete. A second phase of literature search was performed to update the literature until October 2017 using the same search terms.

Once all searches were performed, all titles and abstracts were divided into four alphabetical sections and first screened by pairs of reviewers (with the exception of those written in German which were screened by one reviewer, AD). Of note two literature reviews on fear of cancer recurrence by Simard and colleagues [13] and Crist and Grunfeld [25] included articles up to 2010. Therefore only those articles pertaining to cancer published between 2011 and October 2017 were included in this review. Overall, articles were screened based on the following inclusion criteria: a) published in a peer-reviewed journal between January 1996 to October 2017 (we choose to limit ourselves to the last 20 years of literature to reduce the potential number of articles we would have to review to a manageable amount and to correspond to DSM-IV and DSM 5 literature); b) written in English, French, or German; c) include an adult population; d) report quantitative results on HA-related constructs; and e) include individuals with a chronic illness.

Exclusion criteria consisted of: a) pediatric studies; b) studies with healthy individuals only; c) studies that did not separate data between healthy and chronically ill patients; d) no quantitative data; e) case reports; f) review articles; g) not a peer reviewed article (books, chapters, poster abstracts, conference proceedings, or dissertations); h) editorials; i) irrelevant publications (i.e., not pertaining to HA as defined above); j) language other than English, French, or German; and k) duplicate publications. Inter-rater agreement was obtained based upon consensus between paired reviewers and discrepancies were resolved through discussion. A second screening was performed, in which all retained articles were divided into four alphabetical sections and were read by pairs of reviewers (with the exception of articles written in German which were extracted by one reviewer, AD). The references in identified papers were reviewed for additional relevant articles.

### Data extraction

Data extraction was performed using a standardized data spreadsheet to include the following information when available: a) details of the publication; b) study location; c) study design; d) sample and chronic illness studied; e) definition of the HA construct; f) HA measure; g) prevalence of HA; h) sociodemographic correlates of HA; i) physical and medical correlates of HA; j) psycho-social correlates of HA; and k) interventions. Inter-reviewer agreement was achieved by comparing data extraction between paired reviewers.

### Quality ratings

To assess the quality of intervention and non-intervention articles appropriately, two assessment tools were used. We used a checklist for assessing the quality of qualitative and quantitative studies developed by the Alberta Heritage Foundations for Medical Research [26] for all non-intervention studies. This 14-item scale can be used to rate a variety of quantitative study designs. Studies involving interventions were rated using a checklist for measuring study quality [27]. The checklist contains 27 items that assess validity, bias, and confounders.

### Data analysis

Our aim was to draw comparisons among definitions and measures of HA across chronic illness to establish how this construct is most often defined and measured (i.e., what appear to be its core features across populations). In order to do so, we adopted a multidimensional perspective of this phenomenon and examined if existing definitions and measures tapped into some or all of the following four dimensions: a) affective, or the tendency to be excessively afraid about illness and health; b) cognitive, or the tendency to believe one is ill despite disbelief by others; c) behavioral, or the tendency to seek reassurance for perceived health concerns; and d) perceptual, or the tendency to focus on bodily sensations [11]. When possible, we calculated the frequency of different types of results across studies to describe patterns in the data. For example, we report on different ways of examining prevalence such as using a validated cut-off, percentile score, or percentage of respondents who endorse ‘high’ scores. For the course and correlates, we tabulated statistically significant and non-significant findings. This is a recommended first step in a preliminary synthesis of results and was further subjected to comparison across studies (e.g., not all studies necessarily get the same weight in the final interpretation of the data) [28].

## Results

### Study selection and characteristics

**Fig 1:**
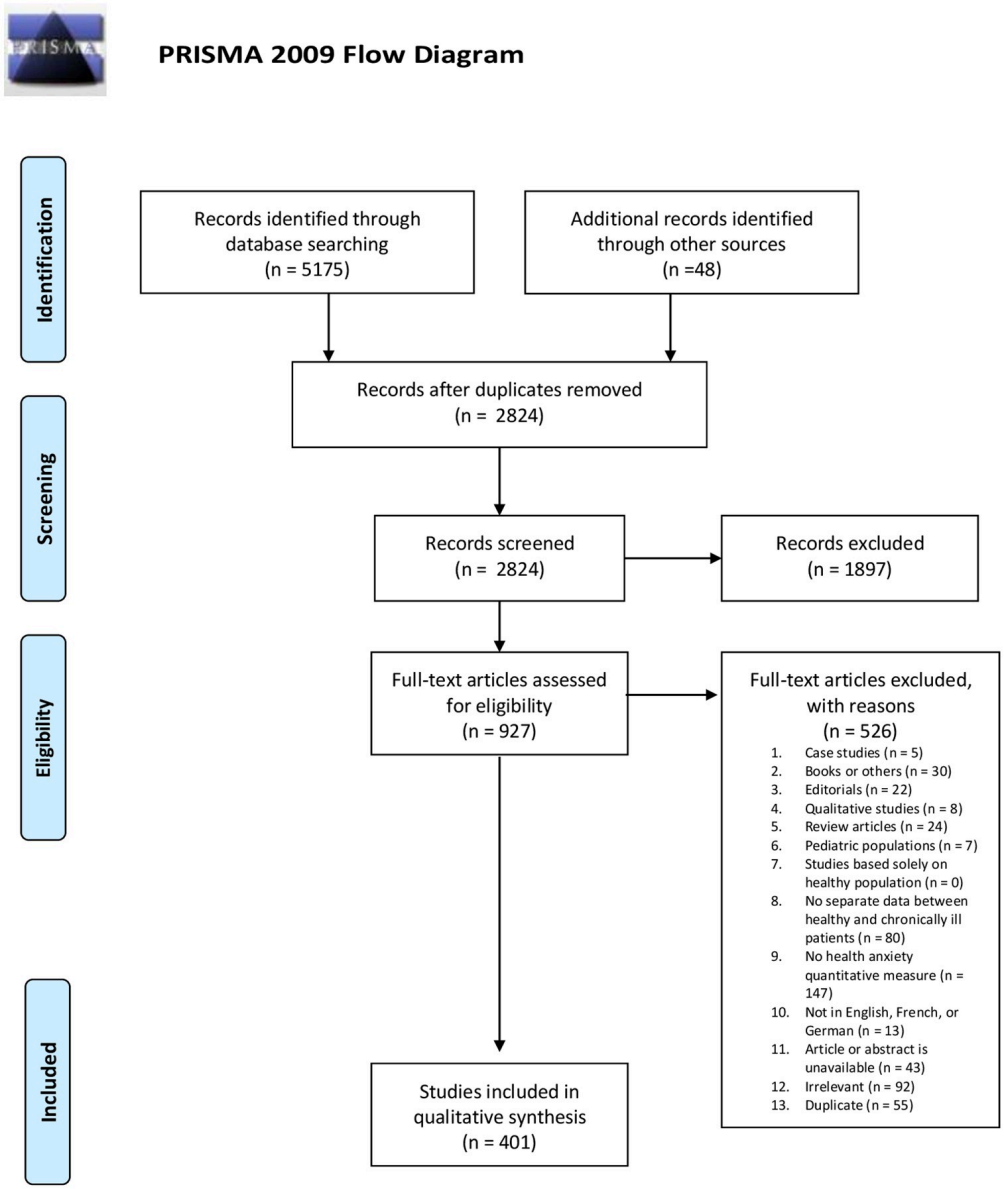
PRISMA flow diagram. *From*: Moher D, Liberati A, Tetzlaff J, Altman DG, The PRISMA Group (2009). *P*referred *R*eporting *I*terns for Systematic Reviews and *M*eta-/Analyses: The PRISMA Statement. PLoS Med 6(7): e1000097. doi:10.1371/journal.pmedl000097 **For more information, visit** www.prisma-statement.org.

Please see Fig 1 for the PRISMA flow diagram of the article selection process. A total of 3229 publications were found at Phase I and an additional 1946 at Phase II. An additional 48 relevant articles were identified by reviewing the references of identified papers. A total of 927 articles were retained after title and abstract screening and were assessed for eligibility, resulting in 401 articles that were retained for data extraction.

Table 2 presents the location, sample size, study design, and type of chronic illness of the 401 articles. The majority of studies were conducted in Europe or North America, had less than 200 participants, used a cross-sectional design, and were conducted with cancer patients.

**Table 2:** Description of studies by location, sample size, study design, and chronic illness (k = 401).

Location			
Descriptor	# Studies	Descriptor	# Studies
**Europe**	160	**Middle East**	12
Germany	45	Israel	8
UK	33	Iran	4
Netherlands	27	**Central/South America**	5
Others	55	Brazil	3
**North America**	145	Puerto Rico	1
**Asia**	34	Peru	1
China	9	**Africa**	3
Japan	7	Nigeria	3
Thailand	4	**Multiple**	12
South Korea	3	**Others**	2
Others	11	**Unknown**	4
**Oceania**	24		
Australia	19		
New Zealand	5		
**Sample Size**			
<50	58	500–1000	28
50–199	183	1001+	31
200–499	101		
**Study Design**			
Cross-sectional	238	Instrument validation	32
Longitudinal or prospective	70	Quasi-experimental	14
Randomized controlled trials	37	Other designs	10
**Chronic Illness**			
Cancer	169	Respiratory diseases	10
Parkinson’s disease	67	Inflammatory bowel disease	4
Type 1 & 2 diabetes	65	Arthritis	1
Heart-related disease	38	Neuromuscular & Gait disorder	12
HIV-AIDS & Hepatitis B & C	15	Multiple conditions	5
Neurological diseases	15		

Finally, a total of 366 non-intervention articles were rated using the manual for quality scoring of quantitative studies. Study scores ranged from 0.29–1, with a mean score of 0.88. The majority of the studies were found to have a high quality of data reporting. Lowest scores were attributable to insufficient information on methodology, patient recruitment/characteristics, and statistical analysis.

The remainder intervention articles (n = 45) were rated using a modified Downs and Black checklist [27]. Study scores ranged from 11 to 23 (28 being the highest score) with a mean score of 17.37. Studies typically had a moderate quality rating, and few had high ratings. Lowest scores were attributed to studies with single-group designs. It is important to note that several studies were not specifically designed to target HA, which limited these quality ratings as intervention studies. A detailed description of the interventions and their impact on HA can be found in Petricone-Westwood et al. [17]. In the present review, we are reporting on the definitions, models, measures, and correlates of HA, if applicable, that were reported in the intervention studies.

## Synthesized findings

### Part I: Conceptual issues

#### Definitions

Few articles defined the HA construct they employed. Of the 401 articles that were reviewed, only 86 (21.4%) cited a definition of the HA construct under investigation (cancer n = 45; Parkinson’s disease n = 14; cardiac disease n = 12; diabetes n = 5; other illnesses n = 10). For studies with cancer patients, the predominant HA construct was fear of cancer recurrence (FCR), defined as “the fear or worry that the cancer will return or progress in the same organ or in another part of the body” [29]. For studies with cardiac patients, authors focused on the construct of cardiac anxiety, defined as fear of cardiac-related stimuli and sensations based upon their perceived negative consequences [16]. For diabetes, studies focused on fear of hypoglycemia (FoH), which was defined as specific worries associated with hypoglycemia (i.e., low blood sugar levels and its accompanying symptoms) and included different behaviors to avoid hypoglycemia [30]. Last, articles on Parkinson’s disease focused on fear of falling (FoF), which is defined as lack of confidence or low self-efficacy to be able to perform activities without falling and a resulting avoidance of these across activities. See Table 3 for a complete description of definitions.

**Table 3:** List of definitions and their dimensions by chronic illness.

Disease	Definitions	Dimensions
**Cancer**	1. Fear or anxiety of cancer recurrence in primary location or its metastasis in other organs [31]	E,C
	2. The fear that cancer could return or progress in the same place or in another part of the body. The degree of concern reported by subjects about the chances of cancer returning at a future time [32]	E,C
	3. The fear that cancer may return or progress in the same organ or another part of the body [33]	E
	4. The fear or worry that the cancer will return or spread in the same organ or to another part of the body [34]	E,C
	5. The fear that the illness will progress with all its biopsychosocial consequences or that it will recur [35]	E
	6. The fear associated with the possibility that the cancer will return or progress in the same place or in another part of the body [36]	E
	7. "Cancer worry" described as fear of future tests, new cancer, and recurrence. "Health worry" described as concerns about death and health [37]	E,C
	8. Health anxiety characterized by excessive fear or worry about ill health, resulting in incorrect belief that one has or is in danger of developing serious disease or medical condition [4]	E,C
	9. Health anxiety involves ruminating about having or developing an illness or having an existing illness worsen, preoccupation with bodily sensations, and behaviour such as seeking reassurance or medical attention [5]	C,P,B
	10. The fear or worry that the cancer will return or progress in the same organ or in another part of the body [38]	E,C
	11. Fear of recurrence is often viewed as a multidimensional phenomenon, including emotional components of anxiety and fear, and a cognitive dimension, including worry, preoccupation and intrusive thought [39]	E,C
	12. Fears of health, worries of future recurrence, concerns that current physical symptoms may signal a recurrence, concerns about developing another type of cancer, or worry about future diagnostic tests [40]	E,C,P
	13. A common form of subjective distress, often involving fears related to the cancer itself, to recurrence and metastasis, to follow-up care and periodic examinations, to relying on strangers for activities of daily living as well as to worry about the future life, disability or death [41]	E,C
	14. An adequate and realistic response to an extraordinary life event, such as the threatening diagnosis of cancer [42]	Could not classify
	15. Worry shares with FOR that both are cognitive behaviors aimed at reducing anxious arousal. FOR is a contextually specific and important worry [43]	C
	16. The degree of concern reported by subjects about the chances of cancer returning at a future time [44]	C
	17. The fear that cancer could recur or progress at the same site or in another part of the body after treatment, FCR manifests along a continuum that ranges from a normal reaction to cancer to a pathologic response associated with dysfunctional behaviours, depressive syndromes, and psychosocial distress [45]	E,B
	18. Health anxiety refers to excessive worry about and preoccupation with illness [6]	C
	19. Fear of progression or, more specifically, fear on the part of patients that their disease will progress and lead to either death or disability [46]	E
	20. FCR is defined as the fear or worry that cancer will return, progress or metastasize. FCR is often conceptualized as a multidimensional phenomenon, including emotional components of anxiety and fear, and a cognitive dimension, including worry, preoccupation and intrusive thoughts [47]	E,C
	21. Fear of the disease recurring or progressing in the same organ or a different area of the body [48]	E
	22. The fear or worry that cancer will return in the same organ or in another part of the body [49]	E,C
	23. The fear that cancer could return or progress in the same place or in another part of the body [50]	E
	24. Fear that cancer could return or progress in the same place or in another part of the body [51]	E
	25. The fear that cancer could return or progress in the same place or in another part of the body [52]	E
	26. Fear that cancer could progress or return in another part of the body [53]	E
	27. The fear or worry that their cancer will return or progress, in either the same organ or in another part of the body [54]	E,C
	28. Worry and concern about recurrence of cancer [55]	C
	29. The fear or worry that the disease will return or progress in the same organ or in another part of the body [56]	E,C
	30. Fear of future diagnostic tests, fear of second cancer, fear of metastasis not defined [57]	E
	31. Worry is repeated thoughts about a particular topic, even though continued thinking may not be helpful, and excessive worry may lead to worse health. In the context of a cancer diagnosis, worry might pertain to cancer recurrence or, in the case of recurrent disease, cancer progression [58]	C
	32. The fear of the disease recurring or progressing in the same organ or a different area of the body [59]	E
	33. Fear of recurrence is often viewed as a multidimensional phenomenon, including emotional components of anxiety and fear, and a cognitive dimension, including worry, preoccupation and intrusive thoughts [60]	E,C
	34. The fear or worry that cancer will come back in the same organ or spread to another part of the body [61]	E,C
	35. Worry over cancer, and in particular, a fear that the cancer will return or progress [62]	E,C
	36. Fear of recurrence encompasses a variety of illness-related fears and is defined as the fear that cancer will recur, progress, or metastasize in the same or another part of the body [63]	E
	37. The fear or worry that cancer will return or progress in the same organ or in another part of the body [64]	E,C
	38. The worry that the cancer will return or progress in the same organ or in another part of the body [65]	C
	39. The fear associated with the possibility the cancer will return or progress in the same organ or another part of the body [66]	E
	40. The worry that cancer will return or progress, and it is one of the most common experiences following cancer diagnosis and treatment, affecting over half of all cancer survivors. Although fear of recurrence can be viewed as a normative response to the possibility of recurrence, it is possible for such fear to become excessive and problematic [67]	E,C
	41. The fear that cancer could return or progress in the same place or in another part of the body [68]	E
	42. Previous research has differentiated two levels of fear of disease progression: the mobilizing and the dysfunctional levels. The mobilizing level is defined as a reasonable response to a real threat over the period of disease diagnostics and treatment and suggests an increase in treatment adherence, resource activation, and use of more effective coping strategies. The dysfunctional subtype of fear of disease progression is, in turn, associated with psychological distress, a decrease in quality of life, and an intense cognitive-affective reaction to disease [89]	E,C,B
	43. The fear or worry that the disease will return or progress in the same organ or in another part of the body [69]	E,C
	44. Fear, worry, or concern about cancer returning or progressing [70]	E,C
	45. The fear or worry that cancer will return or progress in the same organ or a different part of the body [70]	E,C
**Cardiac diseases**	46. Heart-focused anxiety is the fear of cardiac-related stimuli and sensations because of their perceived negative consequences [89]	E,C,P
	47. A specific fear of cardiac-related stimuli and sensations because of their expected negative consequences. Includes a) fear and worries about heart sensations, b) heart-focused attention and monitoring of cardiac-related stimuli, c) cardioprotective avoidance behaviour designed to minimize cardiac symptoms or complications [90]	E,C,P,B
	48. Heart-focused anxiety is the fear of cardiac-related events and sensations due to their presumed negative consequences [91]	E,C,P
	49. A fear of heart-related symptoms and sensations precipitated by perceived negative consequences associated with cardiac related sensations [92]	E,C,P
	50. Heart-focused anxiety or cardiac-anxiety is a specific fear of cardiac-related stimuli and sensations because of their expected negative consequences [93]	E,C,P
	51. Health-related anxiety is a specific type of anxiety that leads to increased worrying about one’s health and the belief that normal bodily symptoms are threatening, harmful and medically serious, despite evidence to the contrary. Cardiac anxiety, on the other hand, is a particular presentation of health-related anxiety that refers to the fear of cardiac-related stimuli and sensations based upon their perceived negative consequences [94]	E,C,P
	52. Cardiac anxiety (CA) is the fear of cardiac-related stimuli and sensations, perceived as negative or dangerous. It is a syndrome characterized by recurrent aversive sensations or chest pain, in the absence of physical abnormalities [95]	E,C,P
	53. After a myocardial infarction specific anxiety symptoms related to cardiac stimuli and sensations may develop. This is known as cardiac anxiety [96]	E,P
	54. Health anxiety is a multidimensional negative emotional state involving cognitive–affective “preparation” focused on bodily signs and symptoms because of their perceived or real negative consequences [9]	E,C,P
	55. The fear of cardiac-related stimuli and sensations based on their perceived negative consequences [97]	E,C,P
	56. The fear of cardiac-related stimuli and sensations based upon their perceived negative consequences [98]	E,C,P
	57. Disease-specific anxiety or cardiac anxiety, a condition characterized by cardiac specific-fear, avoidance behaviors, and excessive cardiac symptom monitoring [99]	E,P,B
**COPD**	58. Disease-specific fears defined by anxiety in face of severe physical symptoms and their consequences; fear of dyspnea, fear of physical activity, fear of progression, fear of social exclusion, and sleep-related worries due to COPD [103]	E
	59. COPD-related anxiety: realistic fears related to symptoms or the consequences of COPD symptoms [104]	E
**Diabetes**	60. Two key components or dimensions of fear of hypoglycaemia (FoH) have been identified: specific worries associated with insulin reaction and the different behaviours to avoid hypoglycaemia [15]	C,B
	61. Health anxiety is experienced when individuals are worried about their health and results in bodily sensations being misinterpreted as more serious and threatening than they actually are [7]	C,P
	62. Fear of hypoglycemia may promote anticipatory compensatory behaviors aimed at decreasing the likelihood of hypoglycemia, including injecting lower than the prescribed insulin dose, increasing caloric intake, and avoiding physical activity [85]	B
	63. Psychological insulin resistance (PIR) is various beliefs and negative insulin-related attitudes such as fear of injections, self-testing, and hypoglycemia, anticipated stigmatization due to insulin injections, and expected hardship from insulin therapy, among other factors [86]	E,C
	64. Health anxiety involves concern about one’s health or about having or acquiring a serious disease [8]	C
**Mixed chronic diseases sample**	65. The fear that the illness will progress with all its biopsychosocial consequences or that it will recur [87]	E
	66. The fear that the disease will progress with all of its consequences [23]	E
	67. Fear of progression: a reactive, nonneurotic fear patients are fully aware of. It is based on the experience of a chronic, life-threatening or incapacitating illness [88]	E
	68. Fear of progression is a reactive, non-neurotic fear patients are fully aware of. It is based on the experience of a chronic, life-threatening or incapacitating illness [24]	E
**Multiple sclerosis**	69. Excessive or inappropriate fear that one has a serious illness based on the misinterpretation of bodily sensations or changes [100]	E,P
	70. Fear of falling which is defined as a lasting concern about falling that leads to an individual avoiding activities that he/she remains capable of performing [101]	C,B
	71. Fear of falling refers to the apprehension felt by an individual with regard to falling during particular activities [102]	C
**Parkinson’s Disease**	72. FoF is a lack of self confidence that usual activities can be performed without falling [71]	C
	73. A constant concern about falling, a loss of balance confidence and an avoidance of activities [72]	C,B
	74. Fear of falling defined as a lack of confidence (low self-efficacy) to be able to perform activities without falling [73]	C
	75. Fear of falling construct described as ongoing concern about falling, a loss of balance confidence, a low fall-related efficacy, or activity avoidance [74]	C,B
	76. Fear of falling construct described as ongoing concern about falling, a loss of balance confidence, a low fall-related efficacy, or activity avoidance [75]	C,B
	77. Fear of falling is a disabling phenomenon common among patients with postural instability and gait disturbances; lack of balance confidence, fear of falling, self-imported restrictions on activities of daily living, especially in relatively challenging situations [76]	E,C,B
	78. FoF can be conceptualised in many ways: diminished perceived self-efficacy in performing a range of activities, avoidance of activity, loss of confidence and as a specific expression of anxiety [77]	E,C,B
	79. FOF is an umbrella term that covers fall-related self-efficacy, concerns about falling, balance confidence, and fall-related activity avoidance [78]	C,B
	80. A lasting concern about falling that leads to an individual avoiding activities that he/she remains capable of performing [79]	C,B
	81. A lasting concern about falling that leads to an individual avoiding activities that he/she remains capable of performing [80]	C,B
	82. Fear of falling can be described as low confidence (low self-efficacy) to carry out activities without falling [81]	C
	83. Reduced self-efficacy at avoiding falls during essential, non-hazardous activities of daily living [82]	C
	84. Low perceived self-efficacy at avoiding falls during essential, nonhazardous activities of daily living [83]	C
	85. Worries about illness, health and injury. This includes worries about ageing linked to health decline. This also includes worries about the impact of poor health on QoL [84]	C
**Stroke**	86. Fear of recurrence, fear of having another bleed, a tendency to catastrophize and misinterpret normal bodily sensations as indicating the onset of another SAH [105]	E,C,P
**Dimensions total (k)**		
Emotional (E)	60	
Cognitive (C)	60	
Perceptual (P)	17	
Behavioral (B)	16	

E = Emotional; C = Cognitive; P = Perceptual; B = Behavioral; FCR = fear of cancer recurrence; FoF: fear of falling; FoH: fear of hypoglycemia; COPD: chronic obstructive pulmonary disease

Overall, across all diseases, definitions were disease- or symptom-specific (n = 78; 91%) with only a minority employing a psychiatric perspective on HA (n = 8; 9%). The articles that used a psychiatric perspective were usually published before the change to DSM-5 and conceptualized the fear as irrational or exaggerated, often based on the misinterpretation of benign physical symptoms [4, 6, 7, 9, 100]. Across disease- or symptom-specific definitions, the emphasis is on the emotional (e.g., fear) and/or a cognitive (e.g., worry) aspect of HA (in 60 of the articles with a definition, each). This is congruent with both of the new DSM-5 disorders, which reflect the presence of anxiety or thoughts/concerns about health or symptoms. However, disease- or symptom-specific anxiety was conceptualized as a realistic [42, 106, 107] and non-neurotic [24, 88], fear in the presence of severe physical symptoms [103], going against the idea that the preoccupation is excessive or disproportionate (criterion B of illness anxiety disorder and somatic symptom disorder). Some authors, however, argue that disease-specific HA manifests along a continuum that ranges from a normal reaction to illness to a pathologic response associated with dysfunctional behaviours, depressive syndromes, and psychosocial distress [36, 37]. This would imply that the fear can become distressing for some individuals with chronic disease (Criterion A of somatic symptom disorder) or can be accompanied by excessive behaviors (Criterion B of illness anxiety disorder and somatic symptom disorder). Indeed, health-related behaviors such as avoidance or body checking were sometimes included in the definition of the disease- or symptom-specific HA (n = 16 articles). Also, indicators such as misinterpreting one’s physical symptoms or catastrophizing about them were included in some definitions (n = 17), which would again be congruent with the new DSM-5 disorders where the individual is easily alarmed about personal health status (Criterion C illness anxiety disorder) or has persistent thoughts about the seriousness of their symptoms (Criterion B somatic symptom disorder).

Thus it seems that the move away from an irrational fear of having a disease in DSM-IV to a preoccupation with having a serious illness (Criterion A of illness anxiety disorder) in DSM-5 results is a much better fit with how HA in conceptualized in people with chronic diseases. All definitions acknowledge the importance of fear/worry/concerns/anxiety about the disease itself or some of its symptoms such as falling in Parkinson’s disease or FoH in diabetes. There are two findings from our results that limit the applicability of the DSM-5 disorders to people with a chronic illness vs. the disease- or-symptom specific approach to HA. The first is a lack of agreement about the “normal” nature of these fears vs. the possibility that these fears may, in some cases, become “pathological”. Indeed, excessive or high anxiety about health and symptoms is a key feature of these psychiatric diagnoses but few of the reviewed articles acknowledged this possibility. Second, while some authors conceptualized HA in chronic illness as a multidimensional phenomenon with affective, cognitive, behavioral, and perceptual dimensions, most definitions did not comprise a perceptual or behavioral component. Furthermore, some researchers wonder if behaviors such as body checking in cancer patients or injecting lower than the prescribed insulin dose in diabetes patients should be part of the definition of HA in this context. They suggest that these behaviors are best conceptualized as maintaining features or consequences [108], the same way safety behaviors, such as carrying anti-anxiety medication with oneself at all times, are maintaining features of panic disorder, not diagnostic features. Thus in the absence of excessive health-related behaviors (Criterion D illness anxiety disorder) in most definitions, there appears to be only a partial applicability of illness anxiety disorder to HA in people with chronic illness. For somatic symptom disorder, a patient can have a mild, moderate, or severe presentation, depending on how many of the following are present: excessive *thoughts*, *feelings*, or *behaviors* related to the symptom. Thus, based on the definitions of FoH or FoF, one could in theory (if there was agreement about what constitutes excessiveness) apply the concept of mild or moderate somatic symptom disorder to patients who have excessive concerns and avoidance of activities related to their symptoms.

Thus it appears that **researchers favor disease- or symptom-specific definitions over psychiatric definitions of HA**. However, there appears to be some applicability of DSM-5 disorders to the experience of HA in patients with a chronic illness.

#### Theoretical models

Next, we wished to examine which theories were commonly used across various chronic diseases to explain the phenomenon of HA in these populations. There were 66 articles that described, cited, or referenced a theory or model to guide their research question(s) and/or the interpretation of their results, representing only 16% of reviewed studies. The three models that were commonly used were Bandura’s self-efficacy theory [109] (n = 8: Parkinson’s disease n = 6; cancer n = 2), Cognitive Behavioral Therapy (CBT) models of anxiety [110] or HA [111] (n = 10: cardiac n = 4; cancer n = 4; diabetes n = 1, neurological n = 1), and Leventhal’s Common Sense Model [112] (n = 7: cancer n = 4; cardiac n = 3).

According to Bandura, individuals will engage in behaviors they believe they can accomplish and avoid those they believe they cannot [109]. Bandura’s theory was used as a relevant framework to explain how an individual’s belief that they can perform certain tasks (e.g., manage their diabetes or perform certain activities without falling) can help them manage their chronic health condition and engage in health behaviors [113, 114] and adopt better coping strategies when faced with uncontrollable and threatening situations [42].

According to CBT models of HA [115], four underlying dysfunctional beliefs can lead to HA: one’s perception of the possibility of experiencing an illness, one’s perception of the consequences of experiencing an illness, one’s perception of the inability to cope with an illness, and one’s perception of the lack of external resources (e.g., availability of medical treatment). These four dysfunctional beliefs will, in turn, negatively influence the interpretation of bodily variations through four cognitive and behavioral processes: increase selective focus on health-related beliefs, increase in somatic monitoring and responses, usage of safety-seeking strategies (i.e., maladaptive coping strategies such as reassurance-seeking or excessive body checking), and increase in affective responses [116]. The CBT model of HA has been especially relevant to studies of cardiac patients, in its adaption to a disease-specific model of HA, Eifert’s cardiac anxiety model [16], which will be described in Part II.

Finally, Leventhal’s Common Sense Model postulates that patients represent their illness according to the following five dimensions: the causes of the illness; the disease label or identity and symptoms they associate with the condition; the curability or controllability of the illness; the timeline and cyclicality of the illness; and the consequences of the illness on the patient’s life. These illness beliefs will influence how patients cope with their illness and their affective reactions. This model also recognizes the maladaptive nature of anxious preoccupation, personal checking behaviors, and over-seeking reassurance from doctors and/or family members [117]. Like the CBT model of HA with cardiac patients, Leventhal’s Common Sense Model has been adapted to cancer patients, specifically Lee-Jones’s model of FCR in cancer patients [117], which will be reviewed in Part II.

Thus it appears that **researchers favor disease-specific models over CBT models of HA developed with psychiatric populations**. However, all the disease-specific models emphasize the importance of decreasing behaviors such as avoidance, reassurance-seeking, or excessive body checking, and increasing the beliefs about one’s ability to cope with the disease or its symptoms, which is congruent with the psychiatric perspective of HA.

#### Measurement

A review of the instruments used to assess HA and illness-related fears revealed several issues. First, in a significant proportion of articles (54 out of the 401; 13.5%), authors did not use a validated measure, instead creating their own items or scale or using a visual analog scale. Most measures lack a validated cut-off score to indicate “excessive” or clinical HA. As a result, researchers frequently resorted to a distribution-based approach, using median or standard deviation, to establish a pragmatic cut-off. In some cases, they classified patients as having HA or not based on their endorsement of the highest possible answer choice (i.e. those who picked 4 or 5 as their answer on a 1–5 Likert scale). Third, we found a high number of measures used (n = 41), with only six of these used with more than one disease population: the Falls Efficacy Scale-International [118], the Health Anxiety Questionnaire [119], the Activities-specific Balance Confidence Scale [120], the Fear of Recurrence Questionnaire [121], the Fear of Progression Questionnaire [23], and the Short Health Anxiety Inventory [122]. This makes comparison of HA across diseases challenging. Last, there was limited evidence of gold standard measures in most chronic diseases: the vast majority of scales (56.1%; 23 out of 41) were used in 1–4 studies and only 17.1% (7 out of 41) were used in more than 10 studies, making comparisons within samples of the same chronic disease difficult.

Overall, this review found that researchers tend to avoid the use of a psychiatric perspective in their choice of HA definitions and theoretical models. Similarly, only 4.7% of studies (n = 19) used measures of HA originally designed for healthy or psychiatric populations such as the Short Health Anxiety Inventory [122] or the Health Anxiety Questionnaire [119]. The vast majority used disease- or symptom-specific measures (reviewed below). The relation between both types of instruments was only measured in eight studies [7, 50, 53, 123, 124]. Of these, six reported correlations among the measures, ranging from *r* = .35 to *r* = .77 [7, 50, 53, 123, 125, 126]. Another study of HA in older cancer survivors using the Assessment of Survivors Concerns also found a moderate correlation between the two subscales of general health worry and cancer worry (*r* = 0.47) [127]. Furthermore, exploratory and confirmatory factor analyses revealed that these two subscales were distinct and showed different patterns of correlations with indicators of anxiety and depression. Thus, while the evidence is limited, it points towards moderate measurement overlap between general vs. disease-specific HA.

Table 4 presents the seven measures that were used in 10 studies or more, including some of their reported psychometric properties. All demonstrated adequate psychometric properties but only four have an established-cut-off score, which is needed to reliably distinguish between individuals with a chronic illness that have an elevated or “clinical” level of HA as compared to those in the more normative range. We examined their content using the affective, cognitive, behavioral, and perceptual dimensions of HA proposed by Longley and colleagues [11]. All seven scales had items that fell under the cognitive domain, asking about thoughts, worry, or concern about the illness or one of its symptoms worsening, progressing, or returning. Four had an affective component, asking about fears and other emotions that accompany the thoughts, concerns, or worry. Only two had items about perception of symptoms leading to worry about the illness and four addressed the behavioral domain, including body checking, reassurance seeking, and avoidance behaviors. Thus, **few measures are designed to reflect the multidimensional nature of HA, few can reliably identify those that present with ‘high’ or ‘excessive’ HA, and few capture the avoidance or reassurance seeking behaviors outlined in the DSM-5.** Also, all of these measures, with the exception of the Fear of Progression Questionnaire, have been used in only one or two chronic disease populations, limiting their generalizability.

**Table 4:** Description of the health anxiety measures that were cited by more than 10 articles.

Name and authors	# of studies	Health anxiety domains	# of items	Subscales	Original validation sample(s)	Psychometric properties
Hypoglycemia Fear Survey [128]	37	Cognitive Behavioral	33	Behaviour Worry	777 adults with type I diabetes	Overall alpha = 0.94
						Behaviour subscale alpha = 0.85
						Worry subscale = 0.94
						Overall test-retest reliability = 0.74
						Test-retest reliability for the subscales = 0.63–0.81
						Evidence of construct, discriminant, and convergent validity
Falls Efficacy Scale-International [118]	21	Cognitive	16	N/A	704 people aged between 60–95 years old	Overall alpha = 0.96
						Test-retest reliability ICC = 0.96
						Evidence of construct and discriminant validity
						Cut-off score: yes
Activities-specific Balance Confidence Scale [120]	24	Cognitive	16	N/A	60 community seniors (aged 65–95)	Overall alpha = 0.96
						Test-retest reliability = 0.92
						Evidence of convergent, divergent, and discriminant validity
						Cut-off score: yes [129]
Cardiac Anxiety Questionnaire [123]	30	Cognitive Emotional Perceptual Behavioral	18	Fear Avoidance Attention	178 post-angiography patients in a cardiology unit; 10 outpatients referred to a behavioral cardiology liaison program	Overall alpha = 0.83
						Evidence of convergent validity
Concerns About Recurrence Scale [29]	15	Cognitive Emotional	30	Overall fear index Health Worries Womanhood Worries Role Worries Death Worries	169 women with breast cancer	Overall fear index alpha = 0.87
						For the Worries scale alpha = 0.89–0.94
						Evidence of convergent validity
Fear of Progression Questionnaire [23]	29	Cognitive Emotional Behavioral	43	Affective reactions Partnership/family Work Loss of autonomy Coping	411 patients (188 cancer patients, 97 diabetes patients, 124 patients with rheumatic disease)	Overall alpha = 0.95
						Subscales alpha = 0.70–0.92
						Overall test-retest reliability = 0.94
						Test-retest reliability for the subscales = 0.77–0.91
						Evidence of discriminant and convergent validity
						Cut-off score (for the short-form version): yes [41, 130]
Fear of Cancer Recurrence Inventory [131]	16	Cognitive Emotional Perceptual Behavioral	42	Triggers Severity Psychological distress Coping strategies Functioning impairments Insight Reassurance	1704 breast, prostate, lung and colon cancer patients	Overall alpha = 0.95
						Subscales alpha = 0.75–0.91
						Overall test-retest reliability = 0.89
						Test-retest reliability for the subscales = 0.58–0.83
						Evidence of construct, discriminant, convergent and divergent validity
						Cut-off score (for the short-form version): yes (68)

#### Summary

**Fig 2:**
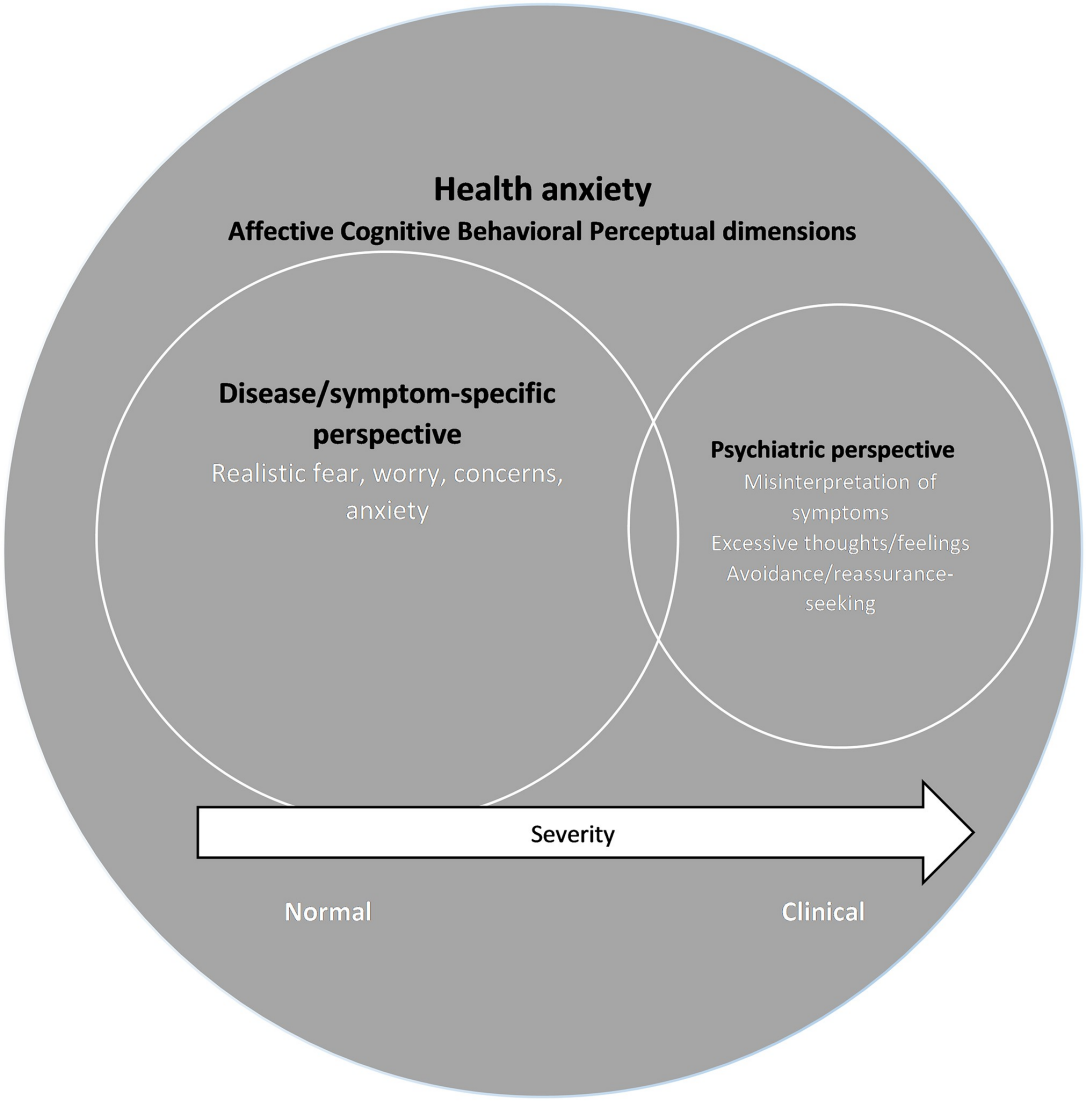
The conceptual relationships between HA, the disease/symptom-specific perspective, and the psychiatric perspective of fears of illness/symptoms recurring or worsening in chronic diseases.

There is modest overlap between the disease- or symptom-specific and the psychiatric perspectives of HA (see Fig 2). Overall, studies used disease- or symptom-specific definitions, models, and measures vs. psychiatric options when conceptualizing HA.

### Part II: Prevalence, course, and correlates of health anxiety in chronic diseases

#### Overall prevalence

Table 5 reports the prevalence of HA across all the reviewed studies. Main results will be presented by chronic disease category below.

**Table 5:** Overall studies reporting on prevalence (%) of health anxiety by chronic illness.

Disease (k)	Mode of determination (k)	Range of prevalence	Author	Reported prevalence	Determination of prevalence scores
**Cancer (58)**		**0–86**			
	Cut-off (20)	4.3–70	Aghdam et al., 2014 [31]	49.6	Scale literature on FoP-Q-SF for “high FoP”
			Anderson et al., 2014 [134]	8	Scale literature cut-off for MAX-PC for “clinical fear of recurrence”
			Butow et al., 2014 [32]	44	Scale literature on FCRI for “clinical fear of cancer recurrence”
			Custers et al., 2013 [135]	51	Scale literature on CWS for "high fear of recurrenc"
			Custers et al., 2014 [33]		ROC curve determing cut-off for high fear of recurrence for the CWS using 2-item CAS
				31	CWS
				40	CAS
			Dinkel et al., 2014 [35]	20	80^th^ percentile on FoP-Q determined by authors
			Ghazali et al., 2013 [36]	35	Established algorithm on 7-item scale identifying “significant fear of recurrence”
			Halbach et al., 2016 [140]	16.9	1 SD above the mean on the FoP-Q-SF
			Hefner et al., 2016 [150]	16.2	1 SD above the mean on the FoP-Q-SF
			Hinz et al., 2015 [137]	16.7	Scale literature cut-off for FoP-Q-12
			Jones et al., 2014 [5]	23.4	Scale literature on SHAI for “clinically signifikant health anxiety”
			Lebel et al., 2013 [38]	58.3	Established in FCRI literature for “clinically elevated fear of cancer recurrence”
			Liu et al., 2011 [61]		Sclae literature cut-off for CARS
				24.8	Moderate
				4.3	High
			Mehnert et al., 2013 [41]	18.1	1 SD above the mean on the FoP-Q-SF
			Petzel et al., 2012 [45]	28–37	Established in FCRI literature for “fear of cancer recurrence”
			Rylands et al.,2016 [156]	23	Highest quartile on seven-item scale
			Sarkar et al., 2014 [47]	36	ROC curve analyses for determination of cut-off for "high fear of recurrence" on the FoP-Q-SF
			Savard & Ivers, 2013 [48]	44.0–56.1	Scale literature for FCRI “clinical levels of fear of cancer recurrence”
			Simard & Savard, 2015 [68]	42	Clinical level of fear of cancer recurrence identified by interview SIFCR
			Thewes et al., 2013 [52]	70	Scale literature for FCRI “clinical levels of fear of cancer recurrence”
	Item severity (23)	0–69.2	Cho et al., 2017 [138]		Items means at “somewhat” or “very much” on ASC subscale fear of recurrence
				36.1	Moderate to high
				13	High
			Cutshall et al., 2015 [147]	52	One item fear of cancer recurrence response "somewhat or a lot bothersome"
			De Padova et al., 2011 [139]		One item fear of cancer recurrence
				58	None/a little
				26	Quite a lot
				16	A lot
			De Vries et al., 2014 [34]		Responses to question "anxious about the possibility that the cancer may return":
				11	Not at all
				74	A little
				11	Somewhat
				0	A lot/very much
			Fang et al., 2017 [148]	48	VAS used to identify "moderate" to "high" fear of cancer recurrence
			Fisher et al., 2016 [149]	50	"Agree" or "strongly agree" on the item "have fear about my cancer coming back"
			Handschel et al., 2012 [146]		Responses to one item on fear of recurrence:
				19.5	Not at all
				30.1	Little
				27.9	Moderate
				16.1	Strong
				6.4	Very strong
			Janz et al., 2011 [151]		Mean on items assessing worry about recurrence:
				14	Not at all
				32	A little bit
				25	Somewhat
				16	Quite a bit
				14	Very much
			Jones et al., 2017 [58]		Response to item about worry about cancer coming back or getting worse
				16.2	Never
				24.5	Rarely
				39.7	Sometimes
				14.7	Often
				4.9	All the time
			Koch et al., 2014 [106]		Established on scale literature of FoP-Q-SF
				82	Low
				11	Moderate
				6	High
			Koch-Gallenkamp et al., 2016 [59]		Established on scale literature of FoP-Q-SF
				87	Low
				9	Moderate
				4	High
			Maguire et al., 2017 [62]		Score standardized 0 to 100
				61.0	Scoring above 25
				18.6	Scoring above 50
			Manne et al., 2016 [63]		Group-based trajectory model using CARS
				25.5	Low-stable
				25.3	High-decreasing
				49.1	High-stable
			Matthew et al., 2017 [152]		Responses to author designed 5-item scale
				36.8–62.2	Not at all
				48.8–51.2	Mild
				34.7–65.3	Moderate
				30.8–69.2	Severe
			Moye et al., 2014 [43]		Indicating "sometimes" to "always" for items:
				44	fear of cancer coming back
				32.5	fear of another cancer
				39	fear of future diagnostic tests
			Myers et al., 2013 [145]	47	"Moderate-to-high" recurrence fears endorsed on CARS
			Ness et al., 2013 [141]		Indicating severity on item on fear of recurrence:
				63	Any level
				17	Extreme
			O’Malley et al., 2017 [153]		Responses on single item on fear of cancer recurrence:
				12.3	Almost never
				17.9	Not very much
				45.9	Sometimes
				23.9	Very often
			Pedersen et al., 2012 [142]		Indicating severity on item on worry about risk of recurrence:
				47.2	A little
				19.3	Quite a bit
				8.5	Very much
			Rogers et al., 2016 [66]		New single item and 7-item scale assessing fear of recurrence
				12	Single item: No fear
				49	Single item: A little fear
				29	Single item: Sometimes having fearful thoughts
				5	Single item: A lot of fears
				5	Singel item: Fearful all the time
				8–23	7-item measure: "a lot" or "all the time"
			Rogers et al., 2017 [155]		Responses on single item measure on fear of recurrence:
				9.3	No fear
				42.0	A little fear
				34.6	Sometimes having fearful thoughts
				9.3	A lot of fear
				4.9	Fearful all the time
				10–19	Responses to seven item scale "a lot" or "all the time"
			Smith et al., 2016 [157]	30	Reporting "very much" or "quite a bit" of fear of recurrence on single item
			Tewari et al., 2014 [143]		Severity of score on frequency of worrying about cancer coming back:
				28.5	Rarely
				19.5	Sometimes
				5.2	Often
				1.9	Always
			Waters et al., 2013 [144]		Indicating on item “worried about progression”:
				69.8	Not at all
				16.0	A little
				10.4	Somewhat
				3.3	Quite a bit
				0.4	Very much
	No cut-off (11)	18–86	Befort et al., 2011 [158]	52	Yes/no to “any fear of recurrence” from checklist of symptoms
			Cheng et al., 2014 [162]	18	Reported unmet need of fears of cancer spreading
			Kanatas et al., 2013 [159]	33	Percentage of clinic appointments when fear of cancer coming back was mentioned
			Posluszny et al., 2016 [154]	86	"Yes"on at least one out of three yes/no items
			Schlairet, 2011 [160]	73.2	Participants identifying “fear of recurrence”
			Scott et al., 2013 [161]	25–52	Self-report for “any fear of cancer recurrence”
			Shay et al., 2016 [67]	85.2/79.7	Yes/no item on fear of recurrence in younger/ older cancer survivors
			Taylor et al., 2012 [49]	67	Minimal possible score of “at least some fear of recurrence”
			Van Liew et al., 2012 [136]	60.1	“Clinically significant levels of fear of recurrence” using FCRI with cut-off not reported
			van Londen et al., 2014 [163]	67	Yes/no fear of cancer recurrence identified as concern
			Wells et al., 2015 [164]	48	Report concerns of fear of cancer recurrence
	Not specified (3)	51–80	Moretto et al., 2014 [165]	80	Unspecified fear of recurrence
			Naidoo et al., 2013 [166]	69	Unclear fear of recurrence
			Pandya et al., 2011 [167]	51	Unspecified feared recurrence
**Cardiac diseases (8)**		**11–48.6**			
	Cut-off (2)	31–48.6	Bunz et al., 2016 [98]	48.6	90th percentile of CAQ score in the general population
			Hoyer et al., 2008 [90]	31	2 SD above the mean of the non-cardiac comparison group for “clinically elevated cardiac anxiety” on CAQ
	Item severity (5)	11–19	Koivula et al., 2010 [171]		"High" fear on CHDPF items for fear of:
				19	Uncertainty about illness
				15	Deterioration of coronary heart disease
				12	Myocardial infarction
			Pauli et al., 1999 [173]		Fear of dying before AICD implantation:
				59	None
				23	Some
				18	Definite
			Rosman et al., 2015 [99]		"Often" or "always" on CAQ items:
				17.6	If tests come out normal, I still worry about my heart.
				14.6	I worry that I may have a heart attack.
			Schuster et al., 1998 [172]		Fear of death identified in concerns list:
				62	None
				18	Slight
				10	Moderate
				3	Quite a bit
				8	Extreme
			van Beek et al., 2012 [96]		Groups identified by latent class analysis
				30.4	Continuously low
				45.4	Continuously medium
				7.7	Continuously high
				16.5	High-decreasing
	No cut-off (1)	16.6	Pollack et al., 2005 [174]	16.6	Clinically relevant heart-focused anxiety on CAQ
	Unspecified (0)				
**COPD (2)**		**14–94.3**			
	Cut-off (0)				
	Item severity (1)	14	Stenzel et al., 2012 [168]		Mean on items assessing worry about recurrence:
				35	No at all
				34	Slightly
				17	Moderate
				14	Strong/very strong
	No cut-off (1)	94.3	Heffner et al., 1996 [169]	94.3	Identified health concern among many concerns
**Diabetes (13)**		**0–81**			
	Cut-off (9)	0–81	Anarte Ortiz et al., 2011 [30]		ROC curve analyses on FH-15 for “fear of hypoglycemia” using single item as criterion
				45.4	FH-15
				48.0	Single item
			Belendez et al., 2009 [15]	33	Unclear cut-off on HFS for "being worried about developing hypoglycaemia"
			Claude et al., 2014 [7]	24.1	Established in literature of SHAI for “elevated health anxiety”
			Cox et al., 1998 [195]		2 SD above published mean in worry scale of HFS
				2	Normally sighted
				0	Partially sighted
				19	Totally blind
			Hajos et al., 2014 [196]		Four criteria to establish cut-off for “clinical fear of hypoglycemia” on HFS worry scale
				81	Modal distribution criterion
				5	SD criterion
				31/21	Concurrent validity criterion
				26	Elevated item endorsement criterion
			Majanovic et al., 2017 [197]	11.1	Establisthed algorithm on elevated item endorsement on HFS-II worry subscale
			Nixon & Pickup, 2011 [198]	27	Score > 50% of total possible score “substantial fear of hypoglycemia”
			Shiu & Wong, 2000 [199]	15	Cut-off > 30 on HFS for "high fear of hypoglycemia"
			van Beers et al., 2017 [204]	25	1 SD above mean in worry scale of HFS
	Item severity (2)	27.7–77	Martyn-Nemeth et al., 2017 [200]		HFS for assessment of "trait fear of hypglycemia" and daily-diary recorded fear of hypoglycaemia
				51	Worry becoming hypoglycemic while sleeping
				77	High daily fear (score 4/5 on two 1–5 items) at least once during six-day study period
			Sakane et al., 2015 [203]	27.7	"Fear of hypoglycemia" was defined as the highest quintile on single item
	No cut-off (1)	30	Myers et al., 2007 [201]	30	HFS "fear of death from hypoglycemia"
	Unspecified (1)	62.9	Riaz et al., 2014 [202]	62.9	Reported “fear of hypoglycemia”
**Epilepsy (1)**		**45.5**			
	Cut-off (0)				
	Item severity (0)				
	No cut-off (1)				
	Unspecified (0)	45.5	Mameniskiene et al., 2015 [205]	45.5	Constant fear of the next seizure
**Hepatitis (1)**		**25–44**			
	Cut-off (0)				
	Item severity (0)				
	No cut-off (1)	25–44	Alizadeh et al., 2008 [194]		Prinicipal concern:
				25	Developing liver cancer
				44	Disease progression to cirrhosis
	Unspecified (0)				
**HIV/AIDS (2)**		**9.4–76.2**			
	Cut-off (0)				
	Item severity (1)		Sarna et al., 1999 [132]	76.2	Indicating "a fair amount" to "very much" on item "worry whether HIV is progressing"
	No cut-off (1)		Kemppanien et al., 2003 [133]		Any indication of "fealing anxious":
				9.4	About the onset of new symptoms
				18.9	About death
**Multiple sclerosis /neuromuscular disorders (4)**		**24.9–63.5**			
	Cut-off (3)	24.9–60.3	Kalron & Achiron, 2014 [102]		Results of ROC analysis of FES-I in another study identifying fear of falling
				39.7	Slightly concerned
				60.3	Highly concerned
			Kehler et al., 2009 [175]	24.9	Scale literature of SHAI for “elevated health anxiety”
			Pieterse et al., 2006 [179]	58	Scale literature of FES for “fear of falling”
	Item severity (1)	63.5	Peterson et al., 2007 [192]	63.5	Yes/no to question "are you concerend about falling"
	No cut-off (0)				
	Unspecified (0)				
**Parkinson’s disease (22)**		**7–89**			
	Cut-off (7)	25–48	Allen et al., 2012 [176]	45.7	Scale literature of FES-I for “high fear of falling"
			Bryant et al., 2014 [71]	44.3	Established in literature for ABC “high fear of falling”
			Chomiak et al., 2015 [177]	25	Scale literature of FES-I for "high fear of falling"
			Franzen et al., 2016 [78]		Scale literature of FES-I for fear of falling
				12	Low
				39	Moderate
				48	High
			Jonasson et al., 2015 [79]		Scale literature on FES-I for levels of fear of falling
				29	Low
				58	Moderate
				47	High
			Landers et al., 2014 [178]	46.4	Cut-off ≥20 on FABQ for "fear of falling avoidance behavior"
			O’Connell & Guidon, 2016 [82]	45.2	Scale literature of ABC for “high fear of falling"
	Item severity (1)	14	Grimbergen et al., 2013 [180]		Single item on fear of falling
				41	No
				45	Somewhat
				14	Very
	No cut-off (10)	35–89	Combs et al., 2014 [181]	44	Yes/no to feeling worried about falling
			Dennison et al., 2007 [182]	35	Yes/no to item “any fear of falling”
			Jonasson et al., 2014 [183]	55	Yes/no to item “fear of falling”
			Kader et al., 2016 [184]	48	Yes/no to item "are you afraid of falling"
			Kataoka et al., 2011 [185]	83	Present or not at any level “fear of falling”
			Lindholm et al., 2014 [73]	37	Yes/no to item “any fear of falling”
			Nilsson et al., 2010 [74]	43	Yes/no to item “fear of falling”
			Nilsson et al., 2010 [186]	38	Yes/no to item “fear of falling”
			Nilsson et al., 2012 [75]	45	Yes/no to item “fear of falling”
			Thomas et al., 2010 [187]	89	At least some fear of falling on FES
	Unspecified (4)	7–83.7	Bloem et al., 2001 [188]	45.8	Any fear of falling indicated in clinical interview
			Cubo et al., 2012 [189]	53	Fear of falling assessed with structured questionaires and ABC
			Lindholm et al., 2015 [190]	83.7	Not specified
			Pasman et al., 2011 [191]	7–15	Not specified: fear of falling during three experimental conditions
**Stroke (3)**		**7.4–56**			
	Cut-off (1)	19	Guan et al., 2015 [170]	19	ABC scale cut-off from validation literature of healthy population
	Item severity (2)	7.4–56	Noble et al., 2011 [105]		Reporting "Extremely fearful" on single item “fear of another haemorrhage”
				7.4	Patients without PTSD
				32.1	Patients with PTSD
			Townend et al., 2006 [193]	56	"Agree"/"strongly agree" on item “worry about my stroke returning”
	No cut-off (0)				
	Unspecified (0)				

In articles that reported longitudinal outcomes, we have reported the baseline findings. Among item severity scores, we have indicated the range of prevalence of highest possible scores.

ABC = Activities-specific Balance Confidence Scale; ASC = Assessment of Survivor Concerns; AICD = Automatic Implantable Cardioverter Defibrillator; CHDPF = Coronary Heart Disease Patients Fear Scale; CARS = Concerns About Recurrence Scale; CAS = Cancer Acceptance Scale; CAQ = Cardiac Anxiety Questionnaire; CWS = Cancer Worry Scale; FABQ = Fall Avoidance Behaviour Questionnaire; FCRI = Fear of Cancer Recurrence Inventory; FES = Falls Efficacy Scale; FES-I = Falls Efficacy Scale–International; FH-15 = Fear of Hypoglycemia 15-item Scale; FoP-Q-SF = Fear of Progression Questionnaire-Short Form; HFS = Hypoglycemia Fear Survey; MAX-PC = Memorial Anxiety Sclae for Prostate Cancer; PTSD = Posttraumatic Stress Disorder; ROC = Receiver operating characteristic; SD = standard deviation; SHAI = Short Health Anxiety Inventory; SIFCR = Semi-Structured Interview on Fear of Cancer Recurrence; VAS = Visual Analogue Scale

#### Cancer

The most frequently cited HA constructs are fear of cancer recurrence (FCR) and fear of disease progression (FoP) (see Table 3 for examples of definitions of these constructs). No single measure emerged as being preferred over others, with the Fear of Cancer Recurrence Inventory, the Fear of Progression Questionnaire, and Concerns About Recurrence Scale all being used in more than 10 studies (see Table 4). Due to the lack of an agreed upon gold standard measure, prevalence rates vary considerably across studies (see Table 5). Even studies that use the same instrument with an established cut-off score report a wide range of prevalence. For example, 28 to 70% of cancer survivors have been identified has having clinical FCR using the Fear of Cancer Recurrence Inventory. In terms of evolution over time, FCR was found to either be stable over time or initially decrease than stabilize [41, 42, 47, 48, 206].

When looking at conceptual differences between cancer-specific HA and other psychological disorders, a moderate degree of overlap emerged. For example, in a study of 341 cancer patients interviewed with the SCID and the Fear of Progression Questionnaire, 17.6% of patients had a diagnosis of anxiety disorder according to the SCID; 68.3% of patients suffered neither from an anxiety disorder nor from FoP; 13.4% suffered only from FoP; and only 6.7% had comorbid anxiety and FoP [35]. In a small study of 60 cancer patients, Simard and Savard [68], also using the SCID, found a slightly higher degree of comorbidity with 16.7% of patients having comorbid anxiety and FCR, mostly with panic disorder and Generalized Anxiety Disorder (GAD). Higher rates of comorbidity were observed in a study of young women with breast cancer using self-reported measures: among those identified as having clinical FCR, 36% were also likely cases of hypochondriasis (measured with a modified Whitely index), 43% were likely cases of GAD, and 20% met criteria for all three [50]. All together, it seems like cancer-specific HA is a distinct phenomenon but demonstrates some overlap with anxiety, especially GAD. Associations with PTSD symptoms [43, 56, 207, 208]; distress [56, 155, 209, 210], depression [39, 53, 63, 68, 106, 155, 209, 211–213], and lower quality of life (QOL) are frequently reported [44, 45, 49, 66, 106, 138, 143]. These relationships are likely bi-directional; for example, FCR predicts future depressive symptoms [211] and previous history of depression is a predictor of FCR [41].

In terms of theoretical model, Leventhal’s Common Sense Model (CSM) and its FCR-specific adaptation by Lee-Jones et al. [117] has been influential. The CSM postulates that FCR occurs when patients encounter an external or internal trigger, which then leads to an increased perception of personal risk of recurrence, both of which have been empirically supported [69, 106, 143, 214, 215]. This model also posits that illness beliefs will influence how patients cope with their illness and their affective reactions. It highlights the maladaptive nature of anxious preoccupation, personal checking behaviors, and over-seeking reassurance from doctors and/or family members [117]. There is good evidence that illness representations predict FCR. For example, in multiple regression analyses controlling for employment, anxiety and depression, illness perceptions, including lower beliefs about treatment control, more negative emotion associated with the diagnosis, longer timelines for the experience of breast cancer, and more symptoms attributed to breast cancer (identity) were significantly associated with FCR [216]. There is also good evidence that coping efficacy [63, 145, 217] and coping strategies [44, 64, 69, 207, 218, 219] influence FCR. For example, women with breast cancer and a pronounced FoP resorted to coping strategies such as “focus on and venting of emotions”, “mental disengagement”, and “behavioral disengagement” to a significantly greater extent than those with lower levels of FoP. They also displayed less confidence in their ability to overcome the consequences of disease and treatment [220].

Other theoretical models appear promising to our comprehension of HA in cancer patients (see Simonelli et al [221] for a comprehensive reviews of different FCR models). For example, social cognitive processing theory [222] can be useful to understand the impact of social interactions on the development and maintenance of HA. Indeed, social support appears to play a role in HA with one longitudinal study of social support at baseline predicting more FCR two years later [61] and additional studies finding that lower social support [4, 41, 140, 145, 223], including holding back [63, 145] correlates with increased FCR. Social constraints showed an indirect effect on FCR through worse cognitive processing (more intrusive thought and more cognitive avoidance). Meta-Cognitive Theory and CBT models of anxiety have also garnered evidence: participants with clinical FCR endorse more positive beliefs about worry, and beliefs about the uncontrollability and danger of worry than those with non-clinical fear [32]. They also report greater anxiety sensitivity [5], body vigilance [5], and the use of reassurance seeking and body checking [52,64,70].

A large body of evidence looked at sociodemographic and medical/physical predictors of HA in cancer patients. Younger age and female gender were consistent predictors of greater HA across studies. There was contradictory evidence for race, education, marital status, and limited available evidence on the relationship between sexual orientation and HA. There is limited evidence of the impact of treatment type such as radiation or chemotherapy on HA [35, 45, 207, 224] but higher levels of HA may influence women with breast cancer to choose contralateral prophylactic mastectomy [225]. Interestingly in the context of personalized medicine and greater treatment options, HA was correlated with concerns about the side effects and long-term effects of aromatase inhibitors [226, 227]. Also, breast cancer survivors who were taking adjuvant endocrine therapy (e.g. tamoxifen) more commonly reported HA than those who have previously and never taken these therapies [163]. The impact of these newer therapies on HA appears to be an area for future research. There is consistent evidence that the presence of symptoms such as pain and fatigue predicts greater HA [33, 41, 45, 126, 142, 160, 226, 228–231]. There is limited evidence that time since diagnosis [35, 38, 41, 207] or stage of cancer [4, 50, 159] correlates with HA. HA was also not related to adherence to surveillance [232] and was inconsistently related with tobacco and alcohol use [54, 233].

HA was correlated with more frequent visits to the ER and outpatient visits to the oncology unit [38], greater use of complementary and alternative medicine and unscheduled visits to their GP but also less frequent ultrasounds or mammograms in the past year [52]. Patients with greater HA felt less satisfied with: a) the information they received [62] b) their care and the medical decisions they made [146, 234], and c) were less confident they had received good treatment [137]. They also reported lower patient activation (i.e., the knowledge, skills, and confidence to manage one’s health) [153] This suggests that the medical team may play an important role in the management of HA in this population.

Of note, two literature reviews specific to studies of FCR up to 2010 were published in 2013 [13, 25]. As such, this literature review included cancer-specific studies from 2011 to October 2017 to prevent duplication. We report many similar findings to the two previously published reviews. Younger age was strongly associated with higher FCR in these previous reviews. One review had also found an association between female gender and higher FCR [13]. Both reviews found that higher FCR was associated with poorer quality of life. As found in the present review, marital status, ethnicity, and education along with treatment and cancer-related characteristics (i.e., stage, time since diagnosis, treatment type) were not generally associated with severity of FCR. Finally, consistent with the present review, experiencing more pain-related symptoms was associated with FCR [13,25], and social support was a predictor of lower FCR [13]. One difference is that the two previous reviews had reported inconsistent evidence supporting the association between psychological factors such as distress, anxiety, and depression and FCR.

#### Parkinson’s disease

The HA construct most often studied among those with Parkinson’s disease is fear of falling (FoF). FoF is typically studied with the Activities-specific Balance Confidence Scale or the Fall Efficacy Scale (see Table 4). There is consistent evidence that FoF is a predictor of QOL [180, 235] and that it may be a stronger predictor of QOL than falls themselves [236]. FoF results in limitations [75, 236], functional impairment [235], and less physical activity [71, 113, 237] or daily activities [238]. There is consistent evidence that people with Parkinson’s disease report more HA than age-matched controls [77, 188, 239–241]. The prevalence of FoF in this population, among articles employing established cut-off scores, is high with reported ranges of 25 to 48% (see details in Table 5). There is a lack of evidence of its evolution over time, with only one study examining changes in FoF over a two week period and finding that it remained stable [183].

Bandura’s self-efficacy theory is the theoretical framework most often cited with this population. As patients with Parkinson’s disease feel less self-confident about performing some non-hazardous activities of daily living, they are less likely to perform these activities [82]. While avoidance of activities due to FoF may reduce the risk of falls in the short term, it is associated with reduced functional mobility and strength and increases the risk of future falls [82].

There is strong empirical support for this proposed bi-directional relationship between functional indicators and FoF. For example, impaired gait or slower gait speed [73, 177, 187, 239, 242–245] predicts greater FoF. On the other hand, FoF predicts current [184, 246] or future avoidance of activities [77, 247] and worse performance on balance or walking tests [73, 181, 239, 241, 242]. This reciprocal relationship may explain why a history of past falls [71, 182, 238, 247–251] predicts greater FoF and why the reverse is also true: FoF predicts more falls 6 [176] or 12 months later [252] or recurrent faller status [253].

Clearly, not all Parkinson’s patients who experience a fall will go on to develop FoF, and while the above factors contribute to the development of FoF, much remains to be discovered about how they interact with each other and over time to lead to FoF [82]. Studies that have taken a multifactor approach to predict FoF show that psychological factors also maintain FoF. For example, 73% of the variance in FoF was predicted by four variables, in order of decreasing importance: walking difficulties (measured objectively with a walking test), fatigue, needing help from others with daily activities, and functional balance [73]. Depressive symptoms and lower cognitive functioning have also shown an association with FoF [72, 78, 187]. More advanced disease stage and longer time living with the diagnosis are associated with greater HA [71–73, 81, 187, 240]. There is limited evidence of sociodemographic correlates of HA in Parkinson’s disease.

#### Cardiac disease

Consistent with other chronic diseases, cardiac patients who report HA display lower levels of QOL [90, 91, 254–256]. In this population, HA is most often conceptualized as cardiac anxiety and measured with the Cardiac Anxiety Questionnaire [123] (see Table 4). Eifert’s cardiac anxiety model has been influential in bringing awareness to the role of catastrophic misinterpretation and attention to cardiac-related sensations and avoidance of activities that induce these sensations (see [89] for a review). According to this model, avoidance behaviors, negative affective states (anxiety and depression symptoms), and increased somatic activity or arousal (e.g. pain) all contribute to maintain HA. There has been empirical evidence of a relationship between these three hypothesized mechanisms and HA in patients with cardiac conditions [90, 94, 96, 123, 171, 254, 256, 257]. In addition, illness representations indicating a more threatening perception of disease, predict greater HA in this population [93, 258, 259].

Patients with cardiac diseases report significantly more HA than healthy controls who are not reporting cardiac concerns [97, 260]. However, there is a vast literature on the differences between HA in people with a cardiac condition and those with cardiac preoccupation such as non-cardiac chest pain [123, 124, 261–263]. While a review of this literature is beyond the scope of this systematic review, it appears that patients with non-cardiac chest pain pay more attention to their symptoms, report more cardioprotective behaviors and greater disease conviction, and view their condition as significantly less controllable and less understandable than those with a cardiac condition [123, 124, 261–263].

In terms of prevalence, 31–48% of cardiac patients were found to have clinically significant cardiac anxiety, according to scale cut-off scores validated in cardiac populations. The evolution of HA is unclear, in part due to different measurement occasions across studies: one study found that HA either fluctuated or decreased over time from 6 weeks post surgery to 6 months later [90], one reported an increase from hospitalization to five years later [264], and one reported that HA was stable over time over a two-month period following a first consultation for chest pain [261]. To better understand these mixed findings, one may also have to consider the patient’s initial level of HA. Van Beek and colleagues [96] found evidence of 4 severity groups among patients hospitalized for a myocardial infarction followed five times over one year, with 7.7% starting with high HA after being hospitalized and remaining high for one year; another group (16.5%) that started high but decreased over the year; and the majority (75.8%) that showed a stable, low to moderate level of HA over time.

There is limited impact of actual disease severity on HA [94, 96, 98] while perceived health [171] and bothersome physical symptoms [171, 259] have been found to predict HA. There have been limited efforts to identify psychological correlates of HA in this population; for example, only one study looked at social support [171]. There is also scant evidence of sociodemographic correlates, and the evidence is inconclusive among the most frequently reported sociodemographic factors, gender and age.

#### Diabetes

In this population, HA is most often conceptualized as fear of hypoglycemia (FoH) comprised of specific worries associated with insulin reaction and the different behaviours to avoid hypoglycemia (see definition above) and measured with the Hypoglycemia Fear Survey [128]. Like other manifestations of HA, it is associated with lower QOL, distress, anxiety, and depressive symptoms [7, 128, 196, 265–271]. Hypoglycemia is a common adverse event for people living with diabetes and can have very severe consequences if untreated, leading to coma and death. Unsurprisingly, studies indicate a high prevalence of this concern (see Table 5). However, there is a wide range of scores, 0 to 81%, due to measurement inconsistencies and the absence of an agreed upon cut-off score on the Hypoglycemia Fear Survey. To illustrate the impact of these measurement issues on prevalence estimates, one study compared four different ways of establishing a cut-off score with the Hypoglycemia Fear Survey and found a range of clinical FoH from 5 to 81% [196]. It appears to be more common among patient with type I vs. type II diabetes [272, 273]. There is a lack of evidence of its evolution over time, with only one study finding that it decreased over a 6 month period [274] and one finding that it remained stable [275].

Early models of the development of FoH suggest that hypoglycemia influences the fear/worry, which in turn triggers avoidance behaviors, which results in poor glycemic control. There is consistent evidence that a greater number of severe hypoglycemia episodes and the presence of more hypoglycemia symptoms are predictors of greater FoH [15, 85, 128, 198, 276]. Similarly, patients who reported needing help to overcome hypoglycemia in the past 6 months [30, 265, 267], difficulty managing their treatment [268], and having a hard time recognising the symptoms of hypoglycemia [277] had greater FoH. Congruent with models of FoH, the presence of avoidance predicts more FoH and hypoglycemia [278]. Patients who report more FoH engage in less exercise [202] and are more likely to restrict their insulin [279]. They also report being more afraid of self-injecting and self-testing [280, 281], and report more issues with insulin management [86] and less treatment satisfaction, which could compromise adherence [282]. However, the link between FoH and metabolic indictors like the HbA1c index is unclear [128, 198, 200].

Since not all patients who experience hypoglycemia will develop FoH, other predictors have been suggested, including the impact of symptoms on everyday life and on daily activities (i.e., not simply their presence) [199]. A link between FoH and trait anxiety has also been reported [15], perhaps because of the tendency to interpret stimuli as being dangerous. Among sociodemographic factors, several studies have reported more FoH in women [7, 128, 196, 265, 272, 283] and there is some evidence of younger patients reporting more FoH [7, 196, 284].

#### Other chronic diseases

Examination of findings across less frequent diseases yielded results similar to those found among patients with more common conditions. For example, patients with Chronic Obstructive Pulmonary Disease (COPD) who report HA also report lower QOL [104, 285], more depression and anxiety symptoms [103, 104, 286–288], and the use of avoidance coping [287]. HA is correlated with less adherence to treatment in HIV patients [289] and may act as a barrier to exercise in seizure patients [290]. As observed with more common chronic diseases, the presence of symptoms or disease-related disability predicts greater HA in patients with HIV [291–293] neurological conditions [175], irritable bowel disease [294, 295], and COPD [103, 288]. Psychological factors such as avoidance of activity [179], causal controllability [193], and social support [175] also appear to play a role in HA in these populations.

#### Summary

HA is prevalent, affecting more than 20% of patients in the studies that we reviewed. There is some evidence of either stability or initial decrease of HA in some chronic illnesses. The presence of disability, limitations, or physical symptoms appears to be a better predictor of HA than more objective indicators of disease severity. There is consistent evidence of the impact of HA on QOL and other indicators of psychological adjustment across diseases. There are few established sociodemographic predictors of HA. Table 6 summarizes the main findings from Part II on prevalence, trajectory, and correlates of HA among patients with cancer, Parkinson’s disease, cardiac disease, and diabetes.

**Table 6:** Summary of main findings on prevalence, trajectory, and correlates of health anxiety among the four most frequent chronic diseases.

	Cancer	Parkinson’s disease	Cardiac disease	Diabetes
**Prevalence**	0–86%	7–89%	11–48%	0–81%
**Trajectory**	Stable over time or initial decrease followed by stabilization	Unclear; limited evidence	Unclear; may depend on patient’s initial level of HA	Unclear; limited evidence
**Correlates**	QOL	QOL	QOL	QOL
	Anxiety and depression	Avoidance behaviors	Avoidance behaviors	Avoidance behaviors
	GAD	Depression	Anxiety and depression	Distress
	PTSD	Lower cognitive functioning	Increased somatic activity or arousal	Anxiety and depression
	Distress			Greater number of severe hypoglycemia episodes More hypoglycemia symptoms
	Internal and external triggers	Physical limitations	Illness representations	
		Functional impairment	Physical symptoms	
	Perceived risk of recurrence	Less physical activity or daily activities	Social support	
	Reassurance seeking and body checking	Impaired gait		Difficulties with disease management
	Illness representations	Slower gait speed		Female gender
	Social support	History of past falls		Younger age
	Female gender	Older age		
	Younger age	Longer time living with the diagnosis		
	Presence of physical symptoms			
	Increased medical costs			
	Satisfaction with care			

## Discussion

Overall, it seems that fears of illness/symptoms represent a relevant field of study, given the large number of studies that were identified for this systematic review. Additionally, the impact of HA on QOL is well documented and there is some evidence that HA predicts: a) lower adherence to treatment; b) fewer positive health behaviours; and c) increased medical costs. Thus this is a very relevant issue for patients, their families, and healthcare providers. One of the most consistent findings of this review is that researchers favor a disease or symptom-specific vs. a psychiatric approach when defining, conceptualizing, or measuring HA in people with chronic diseases. This explains the apparent disconnection between different fields of study, which, in the end, focus on similar phenomena. It seems that the differences in labels (e.g. HA, FoP, FoH), in perspectives (broad vs. narrow), and potentially in professional identities (e.g., psychiatry, psychosomatic medicine, behavioral medicine, clinical health psychology, psycho-oncology) have precluded a unified conceptual view on realistic fears and worries in patients coping with chronic diseases. Therefore, the question remains: how should we conceptualize and name this phenomenon, which seems to be prevalent across many diseases?

One of the conclusions we can draw from this review is that there is a partial conceptual overlap between the current DSM-5 perspective and the way HA is currently studied in the context of chronic disease (see Fig 2). Specifically, the various disease- or symptom-specific definitions had affective (e.g., fear) and cognitive (e.g., worry or concern) dimensions in common, which are consistent with the constructs of somatic symptom disorder and illness anxiety disorder [18]. However, there was no clear consensus about the role of perceptual and behavioral features, which are central to the constructs of somatic symptom disorder and illness anxiety disorder [18]. To this effect, in a recent Delphi study attempting to identify the characteristics of “clinical” FCR, experts did not endorse behaviors such as avoidance or reassurance seeking as key criteria of this construct [296]. This partial overlap is supported by the empirical findings reported in the present review: 1) moderate measurement overlap between general HA and disease-specific HA when studied together, 2) differences between HA in people with a cardiac condition and healthy individuals with cardiac preoccupation, and 3) moderate comorbidity with other anxiety disorders based on structured psychiatric interviews. Thus it appears that worrying about one’s health or symptoms in the context of a chronic illness is somewhat of a different phenomenon than worries in the absence of an illness. Furthermore, in this specific context, we found disease- or symptom-specific HA to be very prevalent, contrary to what has been reported in healthy individuals [20].

Indeed, there is consistent evidence across chronic diseases that HA is prevalent, affecting more than 20% of patients in most of the studied conditions. However, we do not have an agreement on how to distinguish normal from clinical fears and concerns among people with a chronic condition. This, coupled with measurement inconsistencies, leads to imprecise prevalence rates of HA across all the chronic diseases we reviewed. There is some evidence of either stability or initial decrease in some populations but this conclusion remains tentative. The evolution of HA over time may follow different trajectories depending on where patients are along the continuum from more normative responses to clinically significant problems. Those presenting with the highest levels may be more stable which would be in line with DSM-5 conceptualizations. Those in the more normative range may experience fluctuations over time. There is strong evidence across diseases that the presence of physical symptoms is associated with HA, especially those symptoms that are impacting daily living, which is in line with the DSM-5 diagnosis of somatic symptom disorder (Criterion A). However, in the absence of reliable cut-off scores or agreement about what constitutes excessive preoccupation or efforts to deal with these symptoms, the application of this diagnosis relies heavily on clinical judgment.

While it is possible that DSM-5 perspective can apply to the most severe cases, more studies are needed to establish the validity of the DSM-5 formulations of HA in people living with a chronic illness. We recently sought to establish an expert-based consensus on the use of these diagnoses to refer to the most severe forms of FCR. Specifically, we conducted a Delphi study with 65 international experts (psychologists, researchers, nurses, physicians, and allied health professionals) on features of clinical FCR and asked if they would apply the DSM-5 diagnoses of illness anxiety disorder and somatic symptom disorder to patients with high or ‘clinical’ FCR. Only 31% and 25% of respondents, respectively, found these diagnoses applicable to cancer patients with clinical FCR, preferring instead to create and use a list of features that characterize this construct [296]. Further studies are needed to clarify if researchers who study HA manifestations in chronic diseases consider these DSM-5 diagnoses as relevant to their patients’ struggles with worries about their health or symptoms.

In the meantime, based on how the studies included in the present review defined, conceptualized, and measured HA in chronic illness, we think it is best to conceptualize it as HA focusing specifically on the disease or its symptoms that can range from normal to pathological [94]. In our view, the term HA is broad enough to be applicable to diseases with different severity, course, and prognosis. On the other hand, it is specific enough as it implies worries and fears relating to the patient’s health status. We propose the adoption of the following definition of HA because of its multidimensional nature and recognition that the symptoms may, at least in some cases, be reality-based: “A multidimensional negative emotional state involving cognitive–affective “preparation” focused on bodily signs and symptoms because of their perceived or real negative consequences” [119].

Agreeing on a measure that could be used across diseases would strengthen and unify the field. Some of the generic measures of HA have been criticized for their content not being appropriate for individuals with a chronic illness [7, 100, 297]. Based on the present review of the literature, the Fear of Progression Questionnaire [23] appears to be the most promising candidate, since it was developed using a mixed chronic illness sample, has good psychometric properties, uses a multidimensional conceptualization of HA, and has already been used across several chronic illnesses [23, 24, 107]. Furthermore, a valid and reliable unidimensional short form which was developed for screening purposes is available [130, 137]. An empirically derived cut-off score of ≥34 has been suggested to identify those with clinical levels of FoP [47, 88].

Based on the findings of the present review, what indicators can we use to identify patients with severe HA? First, it is important to recognize that there have been few investigations of sociodemographic predictors of HA outside of studies with cancer patients and thus we have very limited evidence of vulnerable populations. Severe HA can be present in any patient, not necessarily those with the most advanced illnesses. However, those with more symptoms, disability, limitations, and impairments in daily functioning are most likely to report high HA. The presence of elevated distress, evidence of sustained use of avoidance or other safety behaviors, beliefs that one cannot cope with these symptoms or disease, and stable course over several months would be indicators of possible severe HA.

### Limitations

Despite our efforts to exhaustively review the literature on HA across chronic diseases, there could be other symptom- or disease-specific terms that describe this phenomenon that were missed, leading us to inadvertently leave out relevant articles. Because we limited the review to articles reporting on HA in individuals with a known chronic condition, we did not include a large number of articles on pain and chronic pain. There are similarities between the activity avoidance pain models and CBT models of HA [104, 286] that would be interesting to explore in future studies. Most included studies (76%) were conducted in Europe and North America and thus our results may be biased towards a Western cultural context. Due to the large volume of articles reviewed, we used several pairs of raters, which may have influenced how systematically our data was reviewed and extracted. We attempted to remedy this by using a standardized data extraction form and consensus discussions to resolve discrepancies. Lastly, the quality of our findings is limited by the content of the articles we extracted data from, many of which were not focused primarily on HA.

### Recommendations for future studies

While our review suggests that there are commonalities in the way HA is defined and measured across chronic illnesses, we need comparative studies of how people coping with different chronic illnesses experience HA. Both qualitative and quantitative designs could shed light on this issue. Studies that compare measures to identify which one best captures HA in chronically ill populations, especially in its most severe form, are needed combined with studies that validate the indicators of severe HA in these populations.

There is also a need for more studies that propose a theoretical framework and validate its use across more than one chronic illness. For example, future studies could validate the components of the Cognitive Behavioral Model of HA developed by Salkovskis and colleagues [111] across chronic illnesses. This would answer the question as to whether this model can be applied to individuals with a chronic illness as it currently stands or if some of its components need to be revised. If the model is found to be applicable, perhaps with disease- or symptom-specific modifications, this could inform interventions that could be transferable across chronic illnesses.

### Conclusions

The experience of fears and worries about the disease or its symptoms returning or progressing is ubiquitous among patients who live with a chronic illness. These fears and worries are often normative in response to living with a chronic disease, but nonetheless can become exaggerated, affecting quality of life and daily functioning. The distinction between psychiatric diagnoses such as illness anxiety disorder or somatic symptom disorder and normal health worries needs to be further studied to minimise the likelihood of ‘over-diagnosing’ and possibly stigmatising health-related concerns in patients with a chronic medical condition. The concept of HA might offer a unifying conceptual perspective on these common fears and worries.

## Supporting information

S1 Checklist PRISMA 2009 checklist. (DOC)

S1 Data (XLSX)
